# Jugular vein distensibility, a noninvasive parameter of fluid responsiveness?

**DOI:** 10.5935/0103-507X.20150039

**Published:** 2015

**Authors:** Glauco Adrieno Westphal, Flávio Geraldo Rezende de Freitas

**Affiliations:** 1 Centro Hospitalar Unimed - Joinville (SC), Brazil.; 2 Hospital Municipal São José - Joinville (SC), Brazil.; 3 Hospital São Paulo, Universidade Federal de São Paulo - São Paulo (SP), Brazil.

Most critically ill patients in intensive care units (ICU) require fluid administration for volume expansion at some point during their hospital stay.^(1)^ In most cases, initial volume expansion does not require more sophisticated or invasive measures. Clinical history data and clinical signs of low flow may suggest the likelihood of a response to the initial fluid infusion. As suggested by Vincent and Weil, “the concept of volemic expansion parallels that of feeding a crying baby who may be thirsty or hungry. The baby’s response to feeding is rapidly apparent as a need is satisfied”.^(2)^

Unfortunately, this basic principle is not frequently used in practice. A recent analysis of more than 2,000 fluid challenges showed that critically ill patients tend to be treated in the same manner, regardless of the initial response to volume expansion. Half of the patients who were responsive to the initial fluid challenge did not receive additional fluid and were subjected to hypoperfusion, and half of the non-responsive patients received fluid and were subjected to fluid overload. In addition, the initial clinical evaluation of the cardiovascular response of approximately 1/3 of the patients was uncertain. Even in these cases, additional fluid tended to be administered to more than half of the patients without a more thorough evaluation.^(3)^ These findings suggest that the fluid challenge frequently depends on a “proof of faith”, which is more strongly based on the belief of the possibility of a clinical response to a fluid challenge than on objective parameters.

It is essential to use monitoring methods capable of quickly and precisely identifying volume deficits to minimize tissue damage related to hypovolemia and avoid iatrogenic fluid overload.^(4,5)^

Several invasive and noninvasive methods, known as dynamic parameters for the evaluation of the cardiovascular responsiveness to volume, have been suggested to improve volume replacement. Among these measures, the respiratory change in arterial pulse pressure (∆Pp) is likely the most well-known method; its first historical reference was in 1669, when Lomer reported a pathological intensification of blood pressure changes in a case of pericarditis, defined by Kussmaul as *pulsus paradoxus* or ‘paradoxical pulse’.^(6)^ In 1899, Otto Frank developed an experimental model consisting of air chambers that simulated the heart-vessel interaction, which helped to define the relationship between arterial tone, stroke volume, and arterial pulse pressure.^(7,8)^ Mechanical ventilation with positive pressure reverses the intrathoracic pressure and increases arterial pressure during inspiration, which was defined as reversed *pulsus paradoxus* in 1973.^(9)^ In 1978, researchers began to evaluate the relationship between the volemic state and systolic arterial pressure variation,^(10-17)^ until 2000, when Michard et al.^(18)^ demonstrated the high accuracy of the clinical use of ∆Pp in the evaluation of fluid responsiveness in septic patients. The pressure changes observed in the arterial bed match those found in the venous bed. Thoracic pressurization acts on the right heart and vena cava, influencing blood return to the heart resulting in changes of the central venous pressure during ventilatory movements.^(19-22)^

In an individual’s responsive to volume, the pressure around the intrathoracic veins (mechanical inspiration) exceeds the internal vessel pressure, and the vascular structure tends to collapse.^(22)^ This constriction generated in the intrathoracic portion of the venous bed during mechanical inspiration functions as a flow resistor, engorging and distending the extrathoracic portions of the great venous vessels, such as the intradiaphragmatic portion of the inferior vena cava (IVC) and the jugular veins. Therefore, responsive patients tend to present with an increase in the inspiratory collapse index of the superior vena cava (SVC) and in the distensibility indices of the inferior vena cava (IVC) and the internal jugular veins during mechanical ventilation.^(21,23)^

In this issue of RBTI, Broilo et al.^(24)^ reinforce the idea that the respiratory variation in the internal jugular vein diameter (ΔDRIJ) is correlated with the respiratory variation in the inferior vena cava diameter (ΔDIVC), suggesting that the internal jugular distensibility may be an easy, noninvasive alternative to evaluate fluid responsiveness in mechanically ventilated patients. IVC imaging can be difficult in obese patients and patients with abdominal distension and ascites, and SVC imaging requires transesophageal echocardiography, which limits its application.^(23)^ Because internal jugular vein imaging does not require transesophageal echocardiography and is technically more simple than visualizing the IVC, this technique seems to be a simple and promising bedside method for the evaluation of fluid responsiveness. However, the limitations of the study should be considered when interpreting the results. Broilo et al.^(24)^ evaluated the correlation between ΔDRIJ and ΔDIVC without testing the capacity of ΔDRIJ to predict fluid responsiveness to volemic expansion based on the cardiac output behavior. In addition, recent studies have questioned the accuracy of ΔDRIJ in predicting the response to volume infusion.^(25,26)^ Thus, as the authors themselves forewarn, the results should be interpreted with caution until new studies are published. We also highlight that the method is applicable for sedated and mechanically ventilated patients. Additionally, data on patients with conditions that lead to an increase in venous pressure (cor pulmonale or ventricular insufficiency) as well as to jugular vein engorgement due to the inadequate position of the head of the bed should be interpreted with caution.^(23,24)^
